# Health status in a transitional society: urban-rural disparities from a dynamic perspective in China

**DOI:** 10.1186/s12963-018-0179-z

**Published:** 2018-12-27

**Authors:** Junfeng Jiang, Peigang Wang

**Affiliations:** 1 School of Health Sciences, Wuhan University, Wuhan, China, No.115 Donghu Road, Wuhan City, 430071 China; 2 Academy of Humanities and Social Sciences, Wuhan University, Wuhan, China, No.299 Bayi Road, Wuhan City, 430072 China

**Keywords:** Self-rated health, Variation rules, Urban-rural disparity, Hierarchical age-period-cohort-cross-classified random effects model

## Abstract

**Background:**

The phenomenon of urban-rural segmentation has emerged and is remarkable, and the health disparities between rural and urban China should be stressed.

**Methods:**

Based on data from the Chinese General Social Survey from 2005 to 2013, this study not only explored the net age, period, and cohort effects of self-rated health, but compared these effects between rural and urban China from a dynamic perspective through hierarchical age-period-cohort-cross-classified random effects model.

**Results:**

Urban-rural disparities, as well as work status and gender disparities in health increased with age, in line with the cumulative advantage/disadvantage effects theory, while marital status disparities in health declining with age was in line with the age-as-leveler effects theory. The war cohort, famine cohort, later cultural revolution cohort, and early reform cohort had poorer health than did those in the early China cohort, economic recovery cohort, and later reform cohort. The economic crisis period, war cohort, baby boomer, and early cultural revolution cohort encountered larger urban-rural health disparities, while the early China cohort and early reform cohort experienced smaller urban-rural disparities in health.

**Conclusions:**

Population health is closely related to social context and health care development. It is necessary to keep economic development stable and boost medical technology improvements and the construction of the health care system.

## Introduction

According to Marx’s theory of political economy, humans are the key factors in the field of production; thus, health plays a non-fungible role in the process of socioeconomic development. To build a comprehensive well-off society, the Chinese government has implemented the national health program, underlining the significance of population health. Additionally, the new medical reform, which aims to handle modern health problems and promote the all-around health of urban and rural residents, has been underway since 2009. Therefore, understanding the factors influencing health accurately and thoroughly is indispensable for constructing a healthy society, and overall knowledge of the health status of a society is a basic precondition, for which longitudinal variations in health are especially pivotal.

With the availability of longitudinal data and the development of multilevel statistical methods over the past several decades, longitudinal analysis methods have entered a relatively rigorous and scientific phase [1]. In fact, longitudinal studies on health have been limited in terms of age and period effects in a long history, and studies on cohort effects of health are limited. However, there is an exact linear dependency among age, period, and cohort (age = period-cohort), which brings about an identification conundrum that has not yet been well solved. Disparities in population health have been mentioned in many studies: for example, most population health studies in the United States have focused on race disparities in health [2–5]. Because of the unique social system, urban-rural disparities have been particularly large in Chinese contexts, and urban-rural disparities in health are worthy of attention and have been examined by some scholars [6–8]. Most studies, however, have been limited to a static perspective or have ignored cohort effects. It is quite clear that the valid identification on cohort effects has introduced a new perspective into population health research with respect to trends and disparities. However, this kind of research is still in its early stages, which needs to be further discussed.

### Urban-rural disparities in health in China

Health disparities that vary with socioeconomic status (SES)-related factors have gained much attention [9, 10]. In recent years, however, increasing numbers of scholars have considered these factors in the context of the urban-rural dual system in China to deepen their analysis. The household registration system, which was formally established in 1958, originally aimed to reduce intra-mobility in China [11], but the current effects of urban-rural segmentation include unfair distributions of various resources [12, 13] such as health care and cultural or educational resources. However, most studies have concentrated on urban-rural disparities in income and the allocation of resources [14, 15], mobility, urbanization, [16] and so forth. Few studies have focused on urban-rural disparities in health and discussed urban-rural health disparities from a dynamic perspective.

In China, most previous studies have noted that the health of urban residents is better than that of rural residents [6, 17]. In cross-sectional studies, many scholars have found that some SES variables, such as education and income, have distinct impacts on health in rural and urban China. For example, Zhao noted that employment had a stronger positive effect for urban residents than their rural counterparts, family income significantly facilitated only rural residents’ health, while education promoted health in rural and urban China equivalently [8]. However, Wang and Cheng argued that only with a higher level of income and education could urban residents have the same marginal increment in health as rural residents do [18]. Certainly, the longstanding urban-rural disparities in health care, social insurance systems, and corresponding resources distributions play a vital role [19]. In addition, some researchers have treated nonphysical factors as the major influencing factors that cause urban-rural health disparities. For example, Dong et al. found that rural residents, compared to urban citizens, received less social support and had more negative emotions, thus leading to poorer health [6]. On the other hand, those researchers who found that rural residents were healthier than urban residents proposed two reasons: first, with greater population density and more severe pollution, urban residents were more likely to experience perceived stress and diseases; second, compared to rural residents, urban residents might have less time to do physical exercise, resulting in a higher morbidity rate for related diseases [7].

### A brief introduction to the age-period-cohort model

Longitudinal studies refer to three temporal dimensions: age, period, and cohort, and specific outcome variables may vary with one, two, or all three temporal dimensions. However, since there is an exact linear dependency among them, period = age + cohort, we usually cannot directly estimate them simultaneously [20–22]. Currently, the age-period-cohort (APC) model, which aims to separate age, period, and cohort effects, is still in an exploratory stage. Nevertheless, some researchers have developed several strategies to disentangle this APC identification problem, such as constrained generalized linear model (CGLM) [23], estimated function methods [24], APC model with intrinsic estimator (IE) algorithm [20], APC characteristic model (APCCM) [25], hierarchical APC-cross-classified random effects model (HAPC-CCREM) [26] and hierarchical APC-growth curve model (HAPC-GCM) [27].

The HAPC-CCREM,^1^ first presented by Yang and Land in 2006 and originally developed to solve the APC conundrum in repeated cross-sectional surveys, combines the data from macro and micro levels well. The HAPC-CCREM has already been applied in longitudinal studies on some popular topics, such as subjective well-being [28] and health [2] in recent years. In HAPC-CCREM, period and cohort effects are both treated as level 2 variables [26]. Different from most previous strategies, the inventors argued that the multilevel design of this strategy could solve the identification conundrum among age, period, and cohort well [22]. They also suggested that the unequal-width intervals setting could be helpful in model identification [29]. However, this strategy has been criticized by other researchers. For example, some evidence shows that the estimation will vary with the width of unequal intervals, because this method is substantially a type of constrained estimator [25, 30]. Other critics argue that the cohort effect produced by HAPC-CCREM will not fit the real data unless the period effect has a nonlinear trend [21, 31]. Despite these cautions, this strategy is useful if researchers can meet certain assumptions that can verify the validity of this unique modeling. For instance, some evidence implies its validity, as there is little distinction between the results from HAPC-CCREM and the results from data generating process, and linear effects can hardly exist in the real world [32].

### Studies on age, period, and cohort effects of health

Despite various difficulties in the identification of age, period, and cohort effects, several APC studies on health have been conducted in recent years. For age effect, the general consensus is that self-rated health (SRH) declines with age, but this change is not linear but reverses to increase among the elderly [29, 33]. However, regarding objective health, the trends are different. Most evidence suggests that overall mortality rate [34] and mortality of various diseases, such as cancer, infectious diseases, and chronic diseases, increase with age [35–37]. There are also many studies on the period effect of health. For example, Salomon et al. and Zheng et al. found that there was an improvement in SRH in the 1980s in the US, while a decline emerged in the early 1990s and a rise emerged again in the later 1990s, followed by a gradual decline in the 2000s [29, 38]. Beck et al., however, found that SRH has continually decreased since the early 1990s in the US [2]. For objective health, the overall mortality rate was found to have a declining trend in the period effect [34], but a rising trend in the period effect of obesity emerged in both China and the US [39, 40]. Other diseases have also shown non-monotonous trends in recent decades [35–37, 41].

Compared with age and period effects, cohort effect has been less examined. Evidence has implied that current health risks of individuals contain the cumulative risks that started from their births, so current health is a kind of cumulative health [34]. Therefore, the potential cohort effect is non-negligible [2, 29]. Many previous studies have discussed cohort effects of health, and most studies have indicated that objective health, such as overall mortality and disease mortality, has a declining trend over cohort [34, 35, 37]. For subjective health, an increasing trend in cohort effect has been identified. For example, evidence from the US has implied that more recent cohorts have better SRH and fewer self-reported physical disabilities [5]. Evidence from China has also demonstrated that earlier cohorts tend to report poorer SRH [42].

As for the population disparities in health over time, scholars have commonly concentrated on how gender disparities [5, 29, 40, 43, 44], race disparities [2, 5, 40], and SES disparities [40, 42] could produce distinct and dynamic impacts on SRH or objective health with age, period, and cohort. Limited studies have discussed urban-rural disparities in health in China. Regarding objective health, evidence has demonstrated that urban residents have higher body mass index (BMI) than rural residents in China, but the BMI gap between urban and rural residents has reduced recently [39]. Regarding subjective health, Chen et al. found that the development of rural residents’ education could eliminate health disparities between cohorts, while the development of urban residents’ education could not do the same in China [42]. Based on the same data, however, Zheng and Zeng argued that urban-rural disparities in health in China were caused by education and income disparities between rural and urban China, and there was no substantial urban-rural health disparity [43]. Although Li and Zhang discussed the urban-rural disparities in age and cohort effects of health specifically, they only identified disparities among the elderly but not adults from all age groups in China [45].

Regarding age effect, there are two competing theories in longitudinal studies on health disparities. One is the cumulative advantage/disadvantage effects theory, and the other is the age-as-leveler effects theory. Cumulative advantage/disadvantage effects theory, also called the Matthew effects theory [46], suggests that the health gap among different social stratifications becomes increasingly larger with age [5, 42, 47]. That is, the gap between those who have advantages in accessing more health resources and individuals who do not have such advantages becomes larger with age. For example, Chen et al. found that the health disparities caused by education brought about cumulative advantages/disadvantages over the course of one’s life: more education brought increasing health advantages with age [42]. On the other hand, the age-as-leveler effects theory states that the health differences among various social stratifications decrease with age [5, 43]. For instance, Zheng and Zeng stated that the health disparities that resulted from SES stratifications in female groups conformed to the age-as-leveler effects theory [43]. In contrast, when discussing variations of health disparities in period and cohort, scholars usually combine the results with specific social events that might be of importance to clearly elucidate these variations. The life course theory, proposed by Glen H. Elder in 1970, can be treated as the theoretical cornerstone of cohort effect. Based on this theory, an individual’s life course is nested in the context in which he/she lives, and his/her behaviors are consequences of social change [48]. Contrary to period effect, it should be noted that with cohort effect, the growth environments have tremendous effects on one’s later life [28]. For example, malnutrition in childhood usually results in poorer health in adulthood [5, 49], thus urban-rural health disparities in childhood can partly explain urban-rural health disparities in adulthood [49].

Based on the summary above, studies on urban-rural health disparities that differentiate age, period, and cohort effects are limited and necessary. Therefore, this paper used the HAPC-CCREM to answer the following three main questions:

1) Were there significant age, period, and cohort effects on health among Chinese residents? If so, how and why did they vary?

2) Were there urban-rural disparities in health in China? If so, how did these disparities vary with age, period, and cohort? What factors contributed to these variations?

3) In what situations could the cumulative advantage/disadvantage effects theory and age-as-leveler effects theory each make sense? What association existed between these two competing theories?

## Data and methods

### Data source

Data for this study came from the Chinese General Social Survey (CGSS) from 2005 to 2013, which contains data from the years 2005, 2006, 2008, 2010, 2011, 2012, and 2013. The cross-sectional survey has been administered seven times, which is in accordance with the basic precondition that longitudinal analysis needs data that are collected across at least three points in time [1]. Launched in 2003, the CGSS is the first comprehensive and large-scale investigation project organized by the National Survey Research Center (NSRC) at Renmin University of China. Since the CGSS2003 contains only urban residents and does not refer to SRH, this study did not use the data in CGSS2003. In CGSS2005, CGSS2006, CGSS2010, CGSS2012 and CGSS2013, 10,000 to 12,500 households were sampled from 400 to 500 villages or urban communities from 100 to 125 counties, districts, or cities randomly distributed in 28 (CGSS2005, CGSS2006, and CGSS2013), 29 (CGSS2012), or all 31 (CGSS2010) provinces for each administration. (For example, in CGSS2013, 100 counties or districts and five big cities were primary sampling units, each county or district included four villages or communities, each big city included 16 communities, and each village or community contained 25 households. One randomly selected respondent in each household completed the survey.) In CGSS2008 and CGSS2011, 6000 and 5620 respondents were sampled from 28 and 26 provinces, respectively. These counties or districts sampled were selected randomly from these provinces that contained eastern developed provinces and middle or western undeveloped provinces, so the data were representative and authoritative. In addition, the history of the CGSS has reflected radical transitions within Chinese society and changes in Chinese behaviors and attitudes [50], because it contains information on family, work and income, lifestyles, social networks, political participation, social cognition, and attitudes, as well as some demographic variables. In this study, after missing data were omitted with a list-wise strategy, the valid sample size was 62,755.

### Variables and encoding

Limited by the inconsistency of most questions in several surveys, some factors that might be significant or were proven to be important were not included in this study. Thus, only gender, age, age-squared, *hukou*, political status, marital status, education, and work status were selected as independent variables at the individual level, and period and birth cohort were selected as independent variables at the contextual level. The former were treated as fixed effects, and the latter were considered as random effects. However, the random effect of *hukou* was also examined in this study. The dependent variable was SRH. Details are presented in Table 1.

**Table 1:** Encodings, means, and notes of variables

Independent variables	Category	Frequency	Percentage (%)	Note
Gender	male female	30,330 32,425	48.3 51.7	
Age				Mean = 46.22 Standard Deviation = 15.48
*Hukou*	urban rural	29,401 33,354	46.9 53.1	
Political status	party member not party member	6608 56,147	10.5 89.5	party is the Communist Party of China (CPC)
Marital status	being married single/divorce/widowed	50,703 12,052	80.8 19.2	
Education	illiteracy primary school junior high school senior high school college or more	8029 14,680 18,919 12,675 8452	12.8 23.4 30.1 20.2 13.5	
Work status	employment unemployment	41,154 21,601	65.6 34.4	unemployment includes jobless, students, and retirement
Period	2005–2013			7 periods
Cohort				15 cohort groups
Dependent variable				
SRH	very poor poor fair good very good	2332 10,484 11,883 22,470 15,586	3.7 16.7 18.9 35.8 24.8	treating as continuous variable approximately

Cohort denotes individuals’ years of birth. In this study, based on data features and the developmental history of China in the twentieth century, we divided cohorts into 15 unequal-width cohort groups: warlord dogfight cohort (before 1925), early country struggle cohort (1925–1929), later country struggle cohort (1930–1934), early anti-Japanese war cohort (1935–1939), later anti-Japanese war cohort (1940–1944), liberation war cohort (1945–1949), economic recovery cohort (1950–1955), great leap cohort (1956–1958), famine cohort (1959–1961), baby boomer cohort (1962–1965), early cultural revolution cohort (1966–1970), later cultural revolution cohort (1971–1976), early reform cohort (1977–1984), city reform cohort (1985–1989), and further reform cohort (1990–1996). Similar strategies can be found in other studies [42] that fit Chinese history better and facilitate an understanding of how social change can have a profound impact on residents’ health. Additionally, this strategy of dividing cohorts on the basis of historical events can make individuals within cohort groups more similar and thus make the variance smaller within cohort groups and larger between cohort groups, which can ensure less biased estimates [30].

The question in the questionnaire that mentioned the dependent variable, SRH, was stated as: “Generally, what is your health status?” Response options consisted of the following: “1=very poor, 2=poor, 3=fair, 4=good, and 5=very good.” Concerning the selection and treatment of the dependent variable, there are two points that need explanation. First, although SRH belongs to subjective health, its predictive power to objective health has proven to be strong; thus, many scholars select SRH as the outcome variable to study population health [42, 43, 51, 52]. Second, it is proven to be clearer and easier to interpret when results are presented with figures and the ordinal outcome variable is treated as a continuous variable. In most situations, these treatments are confirmed to be feasible [29], and the results of the ordinal multi-categorical regression and the general linear regression are similar or almost consistent. Details on period-cohort-specific SRH scores can be seen in Table 2.

**Table 2:** Period-cohort-specific case numbers and SRH scores: CGSS2005–2013

Cohort	Period													
	2005		2006		2008		2010		2011		2012		2013	
− 1924	50	3.280			7	3.143	47	3.319	22	2.500	41	3.000	22	3.046
1925-	161	3.093			16	2.875	114	2.947	69	2.420	115	2.844	82	3.049
1930-	336	3.134			59	2.780	332	2.831	133	2.316	289	2.872	225	2.933
1935-	558	3.249	347	3.213	112	3.098	455	2.886	224	2.469	440	2.843	388	3.023
1940-	560	3.202	625	3.179	308	3.127	587	3.041	291	2.375	599	2.912	513	3.035
1945-	813	3.451	817	3.373	428	3.206	842	3.108	388	2.446	792	3.032	682	3.141
1950-	1012	3.621	1121	3.494	534	3.384	1084	3.235	502	2.518	1101	3.166	993	3.327
1956-	967	3.810	853	3.601	464	3.384	927	3.358	473	2.584	806	3.307	776	3.546
1959-	496	3.881	529	3.630	266	3.489	502	3.478	255	2.714	429	3.385	414	3.630
1962-	1222	4.028	1097	3.736	580	3.676	1203	3.633	487	2.784	1039	3.537	892	3.667
1966-	1383	4.104	1411	3.808	736	3.787	1330	3.754	570	2.918	1217	3.642	1014	3.805
1971-	1240	4.192	1343	3.900	822	3.893	1404	3.944	671	3.006	1266	3.814	1205	3.990
1977-	1209	4.432	1363	4.034	811	4.085	1323	4.174	576	3.198	1257	4.060	1255	4.156
1985-	325	4.640	633	4.103	426	4.270	693	4.293	360	3.419	678	4.252	691	4.301
1990-					59	4.271	302	4.470	197	3.594	486	4.333	586	4.415
Total	10,332	3.878	10,139	3.706	5628	3.682	11,145	3.612	5218	2.821	10,555	3.535	9738	3.709
Mean age	44.697		42.405		43.189		47.292		48.085		48.807		48.524	

### Methods and analytic strategies

Seven administrations of the CGSS produced repeated cross-sectional micro-data, so the HAPC-CCREM was applied to analyze the data. In HAPC-CCREM, period and cohort effects were treated as contextual variables at the same level [26]. Concerning interaction effects, variables at the individual level could influence population health across different ages, periods, and cohorts. In this study, age and other demographic variables, as well as their interaction terms, were added into a fixed effects model, and period, cohort, and *hukou* effects were put into a random effects model. Thus, the model specifications were as follows:

Level 1 model:

$$ {Health}_{ijk}={\alpha}_{0 jk}+{\beta}_1\ast {age}_i+{\beta}_2\ast {age}_i^2+{\beta}_3\ast {gender}_i+{\beta}_{4 jk}\ast {hukou}_i+{\beta}_5\ast {political\ status}_i+{\beta}_6\ast {marital\ status}_i+{\beta}_7\ast {education}_i+{\beta}_8\ast {work\ status}_i+\sum \left({\beta}_m\ast {age}_i\ast {x}_i\right)+{e}_{ijk} $$

Level 2 model:

$$ {\alpha}_{0 jk}={\pi}_0+{p}_{0j}+{c}_{0k} $$

$$ {\beta}_{4 jk}={\pi}_4+{p}_{4j}+{c}_{4k} $$

where *Health*_*ijk*_ denoted outcome variable, SRH; *β*_1_~*β*_8_ were the estimated coefficients of each independent variable: age, age^2^, gender, *hukou*, political status, marital status, education, and work status; ∑(*β*_*m*_ ∗ *age*_*i*_ ∗ *x*_*i*_) denoted the interaction effects between age and previous demographic variables; *e*_*ijk*_ denoted residuals that could not be explained in this model; *α*_0*jk*_ was the intercept, including *p*_0*j*_ and *c*_0*k*_, random effects of period and cohort, respectively, that followed a normal distribution, and the grand mean intercept *π*_0_ after controlling for *p*_0*j*_ and *c*_0*k*_; *π*_4_ denoted the grand mean value of residents’ health after controlling for period and cohort, *p*_4*j*_ was the random coefficient of *hukou* in period *j*, *c*_4*k*_ was the random coefficient of *hukou* in cohort *k*, and these final two terms both followed a normal distribution.

Before the HAPC-CCREM analysis, we centered each fixed effect variable. Centralization has two main advantages: first, it can avoid multicollinearity problems between main effects and interaction effects; second, it can make the intercept value denote the conditional predicted value of SRH after controlling for all other variables in the model. Then, the stepwise regression strategy was used to display the results, and the SAS 9.4 software was used to analyze the data.

## Results

Table 3 shows that in the basic APC model (model 1), the intra-class correlation coefficient (ICC) was 10.04%, indicating that 10.04% of the variation in SRH was explained by period and cohort. In addition, the variance components of period and cohort were statistically significant, suggesting that the utilization of HAPC-CCREM was suitable. The fitness index, Bayesian information criterion (BIC), gradually declined from model 1 to model 5, indicating that the model fitness became increasingly optimized. Gender, age, age-squared, *hukou*, political status, marital status, education, and work status all influenced SRH significantly: being male, urban *hukou*, married, with more education, and employed produced better health. Age impacted health in a nonlinear way, declining gradually at first and then remaining stable or even increasing later. It is important to note that in random intercept models (model 2 and model 3), *hukou* had a significant impact on individual health. However, when *hukou* was added into the random effects models (model 4 and model 5), the impact of *hukou* on health in fixed effects models declined dramatically but remained statistically significant. Therefore, the *hukou* system may have a structural effect, and it is vital to consider its contextual effects on population health.

**Table 3:** HAPC-CCREM analysis of SRH among Chinese residents: CGSS2005–2013

	Model 1	Model 2	Model 3	Model 4	Model 5
Fixed effects					
intercept	3.5909***	3.5056***	3.5019***	3.5021***	3.5017***
age	−.0224***	−.0214***	−.0225***	−.0215***	−.0225***
age squared	.00001	.00026***	.00030***	.00027***	.00029***
gender (ref. male)		−.1077***	−.1160***	−.1090***	−.1159***
*hukou* (ref. urban)		−.0569***	−.0652***	−.0672^+^	−.0722*
politics status (ref. no)		.0592***	.0505***	.0515***	.0487***
marital status (ref. single/divorce/widow)		.1232***	.1180***	.1187***	.1182***
education (ref. illiteracy)					
primary school		.1453***	.1354***	.1407***	.1377***
junior high school		.3227***	.3070***	.3124***	.3084***
senior high school		.3759***	.3688***	.3698***	.3699***
college or more		.3932***	.4016***	.3966***	.4028***
work status (ref. no work)		.1821***	.1887***	.1888***	.1887***
age*gender			−.0009^+^		−.0010^+^
age*hukou			−.0032***		−.0025**
age*marital status			−.0039***		−.0038***
age*work status			.0021**		.0021**
Random effects-variance components					
Period effects					
intercept	.1038*	.1009*	.1008*	.1017*	.1015*
*hukou*				.0046^+^	.0044^+^
Cohort effects					
intercept	.0150*	.0020	.0010^+^	.0016	.0010^+^
*hukou*				.0032*	.0014^+^
ICC	.1004	.0906	.0898	.0972	.0951
Index of fitness					
BIC	182,170	180,307	180,284	180,246	180,243

***P<.001,**P<.01,*P<.05,^+^*P* ≤ .1

### Net age, period, and cohort effects of health

**Fig. 1:**
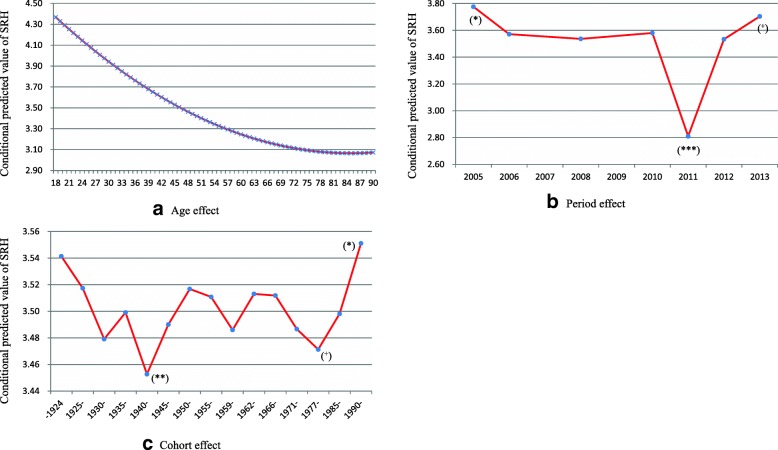
Age, period, and cohort effects of SRH among Chinese residents: CGSS2005–2013. **a**: Age effect; **b**: Period effect; **c**: Cohort effect

After controlling for all other variables in model 5, we obtained net age, period, and cohort effects of Chinese residents’ health. From Fig. 1a we can see that the SRH declined with age, but the rate of decline slowed gradually until 84 years old, at which time the SRH began to improve. For period effect, the SRH of Chinese residents was best in 2005 (*P <* 0.05), then slowly deteriorated from 2005 to 2006, and remained stable from 2006 to 2010. There was a period of declining health in 2011 (*P <* 0.001), after which the SRH rose again rapidly and reached the second highest value in 2013 (*P <* 0.1).

Regarding cohort effect, there were various wars before 1949, and cohorts during this time experienced relatively poorer health; the early 1940s cohort had the poorest health (*P <* 0.01). A general promotion of health emerged from the 1945 cohort to the 1969 cohort, which showed a beneficial effect of the peaceful context on health. There was, however, a small era of decline in the famine cohort. After the later 1960s, a decline in health emerged again, and the early reform cohort experienced poorer health (*P <* 0.1). However, after 1984, residents’ health improved again, and the further reform cohort was the healthiest (*P <* 0.05).

### Urban-rural disparities in health from a dynamic perspective

**Fig. 2:**
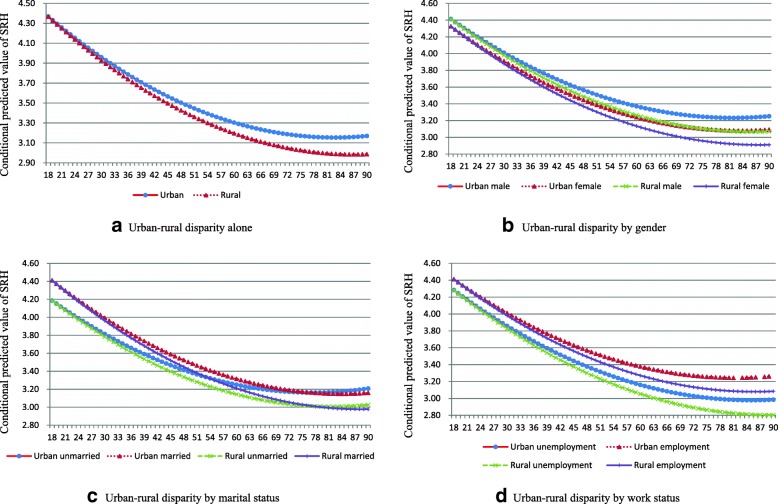
Age effect of urban-rural health disparity in China: CGSS2005–2013. **a**: Urban-rural disparity alone; **b**: Urban-rural disparity by gender; **c**: Urban-rural disparity by marital status; **d**: Urban-rural disparity by work status

Figure 2 shows that the urban-rural health disparities were not constant but varied with age. Before 30 years of age, the difference in the conditional predicted value of SRH between rural and urban China was negligible. After age 30, however, the social health advantages of urban residents emerged gradually, with an accompanying strong cumulative advantage effect.

In the comparison of urban-rural disparities in health by age, gender, marital status, and work status were observed to have significant moderating effects, while education and political status did not have (these two non-significant moderating effects were not reported in Table 3). The SRH of males was better than that of females across the life span, and this gender gap expanded gradually with age. From the perspective of an urban-rural comparison, the cumulative effects of gender disparities in health made sense. With cumulative effects of both urban-rural and gender disparities in health with age, the urban-rural and gender disparities in health increased. In old age, urban males were the healthiest, followed by urban females and rural males (with a small difference between them), and last were rural females. Marital status disparities in health with age showed a converging trend that followed the age-as-leveler effects theory. Individuals who were married had better health than others did in their young and middle age, but this difference disappeared gradually with age; married individuals over the age of 75 had poorer health than did those who were not married. Therefore, considering the urban-rural disparities, both cumulative advantage/disadvantage effects and age-as-leveler effects existed in the urban-rural and marital status disparities in health with age. The variation of SRH by work status with age showed a similar trend as with gender in the context of an urban-rural comparison. With the double impact of cumulative advantage/disadvantage effects of urban-rural and work status disparities, the urban-rural health disparities enlarged with age in China.

**Fig. 3:**
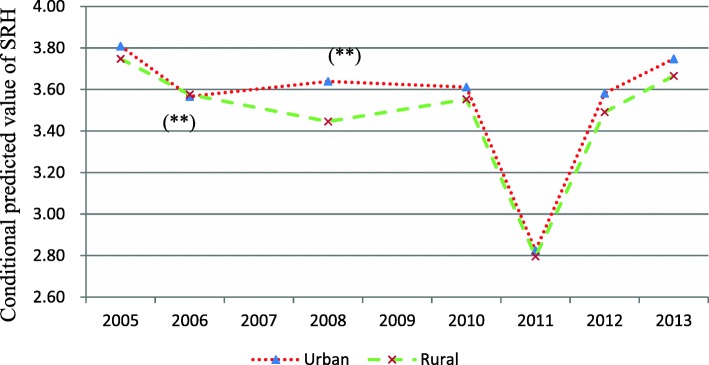
Period effects of urban-rural health disparities in China: CGSS2005–2013

In model 5 and Fig. 3, we identified not only age variations in urban-rural health disparities, but also period and cohort variations in urban-rural health disparities. The health of urban residents was better than that of rural residents globally, with only one exception in 2006 (*P* < 0.01). The difference in the conditional predicted value of SRH between rural and urban China reached its peak in 2008 (*P* < 0.01) and declined in later years.

**Fig. 4:**
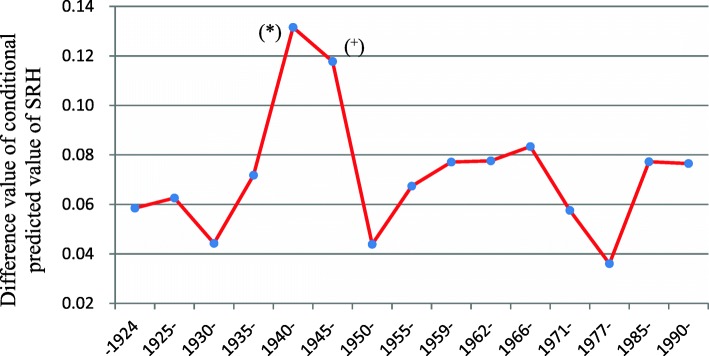
Difference value of cohort effects of urban-rural health disparities in China: CGSS2005–2013

The cohort effect of urban-rural disparities in health presented similar trends, with some discrepancies across cohorts. Subtracting the conditional predicted value of SRH in rural China from that in urban China produced Fig. 4, from which we could more clearly recognize that the urban-rural disparities in health varied with cohort in historical contexts. There were three high and three low periods in urban-rural health disparities during the twentieth century. The decline occurred with the later country struggle cohort, early China cohort, and early reform cohort, and the increases occurred with the 1940s cohort, the 1960s cohort, and the after 1985 cohort. The urban-rural disparities in health of the 1940s cohort were the largest (*P* < 0.05), while the early reform cohort experienced the smallest urban-rural health disparities.

## Conclusions and discussion

Based on data from the CGSS from 2005 to 2013, this paper analyzed the age, period, and cohort variations in health among Chinese residents through HAPC-CCREM as well as the dynamic comparisons of urban-rural disparities in health on these three temporal dimensions. Two main discoveries can be observed. First, there were significant age, period, and cohort effects of health among Chinese residents. There was a curvilinear association between health and age. Although health became poorer with age, there was a “selective effect” among the elderly that made health improve. The period and cohort effects of health were influenced by related social events, war and social instability worsened population health, while peace and health care development improved population health profoundly. Second, the urban-rural disparities in health varied with age, period, and cohort dynamically. Based on cumulative advantage/disadvantage effects, the urban-rural health disparities increased with age. This trend showed double cumulative effects when examining gender and work status disparities, and both the cumulative advantage/disadvantage effects and age-as-leveler effects when examining marital status disparities. For period and cohort effects, the urban-rural health disparities might be impacted by health care systems and policies, as well as the stability of the social context.

As for age effect, the urban-rural health disparities emerged to be significant after the age of 30, which suggested a difference in social insurance between urban and rural China. Residents could stay healthy due to the health advantages of youth. After age 30, however, with the decline of the biological advantages of youth, social insurance factors played a more vital role. Since socioeconomic and health insurance in urban China were better than in rural China, and urban residents’ health literacy was also higher, the urban-rural disparities in health became increasingly larger with age according to cumulative advantage/disadvantage effects. However, the cumulative advantage/disadvantage effects theory and age-as-leveler effects theory were observed not to be competing substantially. In the comparison of group disparities with age, the cumulative advantage/disadvantage effects theory was applicable to characteristics that could bring material resources, while the age-as-leveler effects theory was suitable for features that produced affective resources. Cumulative advantage/disadvantage effects might transform into age-as-leveler effects gradually with the infiltration and increase of affective factors. In this study, education and work were two vital approaches to gain economic resources, and urban-rural segmentation was reflected to a great extent in the disparities in educational attainment and work status [13], where urban residents had more advantages than rural residents. This led to the cumulative advantage/disadvantage of health with age. The trend of health disparities between married individuals and others with age could be interpreted by age-as-leveler effects theory well, which could largely be accounted for by the affective components of marriage, for maturity or affection with age could eliminate the original health disparities [5]. Generally, females were observed to be more affectionate than males, so the health disparities caused by gender had intra-gender differences, and the age-as-leveler effects on health disparities were more evident among females [43]. Therefore, this study offers a proposition about these two theoretical perspectives: the cumulative advantage/disadvantage effects will transform into age-as-leveler effects gradually with the infiltration of or increase in affective factors. Certainly, this proposition needs to be examined and discussed further.

As for period effect, there was a period of declining health in 2011. In fact, although there was a severe financial crisis in 2008, the H1N1 influenza virus broke out in 2009, and the new health care reform was implemented in 2009,^2^ no significant inter-period variation was observed before 2010. Since the health outcome was SRH, a subjective health index, the remarkable decline in 2011 might be partly due to the Fukushima nuclear disaster in Japan in 2011, which resulted in some psychological panic among Chinese residents.^3^ Furthermore, Chen and Wang’s research also indicated that the mortality rate of urban residents in China during 2010–2014 was slightly higher than during 2005–2009 [34], which also suggested a strong association between subjective and objective health.

The health of urban residents was better than that of rural residents globally, which was in line with previous studies. The difference in the conditional predicted values of SRH between rural and urban China reached its peak in 2008, with a decline in later years. The new health care reform that started in 2009 might have played a vital role in coordinating health care resources and guaranteeing rural residents’ health through the equalization of health care resource distribution between rural and urban China in actual operations or through the psychologically motivating effect on the subjective health of rural residents brought on by the reform itself since SRH reflected individuals’ perceptions of their health but not actual health-related behaviors or objective health outcomes [52]. However, because of various problems that appeared in the new health care reform, there was a trend of widening urban-rural health disparities after 2011. The previous psychologically motivating effect receded or vanished with period, and people evaluated their health based on the actual effects of the reform. Since the rationalization of a health care policy could be evaluated through its effects on population health [53], this phenomenon also implied that the new health care reform still needed to do more with regard to the equitable distribution of health care resources between rural and urban China.

Since the period effect discussed above was not linear, we believed that the cohort effect was reliable in this study [31]. For cohort effect, a turbulent social context could greatly damage population health, while a peaceful social environment with a stable social order was the most reliable insurance for population health. Before the establishment of new China, there were various wars, which gave rise to an abominable living context for these war cohorts. In addition, psychological traumas, limited education, malnutrition, and poor living conditions in childhood produced poorer health in later years [5]. After the establishment of the People’s Republic of China (PRC), a promotion of health that reflected the facilitation of the peaceful environment emerged. There was, however, a small period of decline in the later 1950s and the early 1960s, which could be reasonably elucidated by the Great Chinese Famine. The damages of the Great Chinese Famine from 1959 to 1961 to health were mainly embodied as malnutrition and abominable living conditions. According to “selective effect,” those who could survive during that period were healthier or had stronger willpower than others that could not survive. This famine occurred in a relatively peaceful environment, so its negative effects on population health were not as profound as we expected [54]. In addition, the Baby Boomer was proved to be unhealthier in some other studies, for there was more stress of social competition among the baby boomers [5]. This effect, however, did not emerge significantly in this study. After the later 1960s, China witnessed the Great Cultural Revolution and Reform and Opening-Up. As a major mistake in the process of national development, the detrimental impacts of the Great Cultural Revolution on China were not limited to politics but were also seen in the economy, health care, education, and other areas. Since the existence of these adverse living conditions, individuals belonging to this cohort did not have adequate education and nutrition, resulting in poorer health. The Reform and Opening-Up was a profound social-economic transition, but it also brought about adverse impacts on social development in its early phase. For example, in the early era of the reform, the old health care system was damaged, but new health care system had not been constructed, thus leading to some limits to health care development. Additionally, the introduction of market-oriented approaches in hospitals made it more difficult for poor children to obtain health care services. However, since this transition emancipated productive forces and optimized social structures, the detrimental influences on health were neutralized by its benefits, so the health of Chinese residents improved after 1985.

There were three high and low periods in urban-rural health disparities during the twentieth century. In the later 1930s and 1940s, the urban-rural disparities in health rapidly grew and reached a high point. This was mainly caused by the destruction of the Anti-Japanese War and the War of Liberation on Chinese society, and rural cohorts faced more health challenges due to their fragility in times of war. In the early 1950s, the establishment of the PRC and the valuable peaceful social environment provided immense insurances for socioeconomic and health care development. In addition, the three-level network of medical treatment in rural China in the 1950s [55], which was the beginning of the rural cooperative medical care system, supplied a systemic foundation for a reduction in the urban-rural health gap, and this health care achievement was shared by the rural cohorts at that time. The urban-rural disparities in health appeared to grow again in the later 1950s and the early 1960s. Owing to policy weaknesses with respect to social development, the Great Chinese Famine that occurred in 1959 damaged the rural health care system substantially, and millions of people starved to death. Health care development, especially in rural areas, nearly stopped. In addition, limited food and health care damaged the health of rural cohorts profoundly. In the 1970s, based on the development of barefoot doctors in the 1960s and the call of the central government that put the center of health care into rural areas [55], urban-rural health disparities reduced again and reached a low point in the early 1980s. With the implementation of the reform and opening-up policies, most health care resources flowed to urban China under the market mechanism, and urban-rural health disparities increased again. However, these disparities did not become larger but remained stable in the later reform cohort. Although China has conducted a market-oriented reform, the government still occupies a leading position in most key fields and can make some proper resource redistributions. For example, due to the operation of medical insurance and the one-child policy, people in rural China could bring up children with more resources and ensure the nutrition and health of their children more easily; thus, the cohort effect of urban-rural health disparities was gradually reduced [49, 56].

The results clearly demonstrated that a stable social context and sufficient health care resources were indispensable to improve population health. Other studies, however, presented distinct views. They found that a war context made individuals more positive and optimistic, while recent cohorts were more unhealthy because of more social stress [5]. The impacts of social developmental stages and social systems may matter.^4^ For example, Yang et al. argued that the baby boomers would experience poorer health, and the health disparities would decrease with cohort in the US [5], but this was not apparent in the present study. They explained that the baby boomers would experience more drastic competitions when they entered the labor market, thus leading to poorer health. Compared to the US, however, China was in a distinct developmental stage, and the baby boomers of the 1960s entered the labor market in the 1980s or 1990s, during which the labor demand was huge. Since labor-intensive industry became dominant in the later twentieth century in China, the competition effect was greatly reduced by the positive effect of economic benefit from large labor demand. On the other hand, as a comprehensive institutional system that has existed about 60 years, the *hukou* system has become deeply embedded in Chinese residents’ daily lives. It brings about unequal distributions of health resources and socioeconomic development between urban and rural China, which causes the urban-rural disparities in health. Its limitations have emerged gradually in the currently changing social context because of its relative-solidified framework, leading to the failure of many other policies on the balanced allocation of various resources between rural and urban China. Therefore, reform to the *hukou* system that can reduce inequalities caused by the *hukou* system is necessary for the operation of other policies to motivate the equalization of urban-rural development.

This study still has some limitations. First, as an institutional segmentation tool, the Chinese *hukou* system has resulted in urban-rural disparities in many areas, including social insurance and health care. Thus, examining how these differences lead to distinct health outcomes is valuable but challenging. Then, with the increase in internal migrants, the segmentation of *hukou* system not only emerges in urban-rural areas but also appears as a unique internal division in urban China. This brings about a new dual system in intra-urban areas [57], but we did not discuss this phenomenon in detail. In addition, this paper used only a subjective health indicator as the health outcome to examine age, period, and cohort effects and urban-rural disparities in health, and objective health indicators need to be discussed, if possible. Finally, according to previous studies, the HAPC-CCREM we used still cannot solve the APC identification problem well. Although we have taken several measures, such as unequal-width intervals of cohort groups based on Chinese history, to make the results reliable, we should treat the results in this study with caution. These limitations point to further research topics in the field of population health, and our research is just a beginning for related studies.

## Abbreviations

APC: Age-period-cohort
APCCM: Age-period-cohort characteristic model
BMI: Body mass index
CGLM: Constrained generalized linear model
CGSS: Chinese General Social Survey
CPC: Communist Party of China
HAPC-CCREM: Hierarchical age-period-cohort-cross-classified random effects model
HAPC-GCM: Hierarchical age-period-cohort growth curve model
ICC: Intra-class correlation coefficient
IE: Intrinsic estimator
NSRC: National Survey Research Center
PRC: People’s Republic of China
SES: Socioeconomic status
SRH: Self-rated health

## Footnotes

1 We discussed only the HAPC-CCREM, because it was the method we used to analyze the data.

2 The New Healthcare Reform, which started in 2009 and is currently in effect, has had profound effects on the medical system in China. It sets a guiding ideology, basic principles, and a global purpose of the health care reform, and especially stresses the equality of urban and rural areas in health care services allocation.

3 In another study conducted based on the World Value Survey, we compared the SRH variations among China, Japan, Russia, and the US with the IE algorithm. We found that SRH deteriorated greatly among Japanese residents in the early 2010s, the health of Chinese residents worsened only slightly, and no significant decline in health was found among Russian and American residents. Thus, we speculated that health-related international events would influence health abroad through a specific spatial model, with those in closer proximity being impacted more significantly. Certainly, the low level of SRH in 2011 might also have been caused by sample bias or selection bias.

4 Some more details can be seen in Chen et al.’s study.
